# Esophageal foreign body removal under holmium laser-assisted gastroscope: A case report

**DOI:** 10.3389/fsurg.2023.1094160

**Published:** 2023-01-17

**Authors:** Zhe Yang, Shanyu Qin, Xiaomin Li

**Affiliations:** Department of Gastroenterology, The First Affiliated Hospital of Guangxi Medical University, Nanning, China

**Keywords:** esophageal foreign body, foreign body removal, holmium laser, case report, thoracic esophageal

## Abstract

As a common clinical emergence, esophageal foreign body can lead to esophageal perforation followed by severe complications including aortic injury, mediastinal abscess and airway obstruction, leading to a high rate of mortality. Therefore, fast and effective diagnosis and treatment are of great necessity. In this case, holmium laser-assisted gastroscopy was adopted to remove the foreign body incarcerated in the esophagus, allowing patients to avoid traumatic and costly surgeries. It is a supplement to traditional methods of foreign body removal. The new combination tried in this report can bring development and innovation inspiration to the development of endoscopic technology.

## Introduction

As a common clinical emergence, esophageal foreign body can bring about various symptoms such as dysphagia, chest pain, vomitting, cough and dyspnoea (1). The common types of foreign bodies incarcerated in the esophagus include fishbone, bones of other animals, date pits, plastic or metal products, dentures, etc (2). In cases with esophageal foreign bodies, repid diagnosis and timely treatment are required. Apart from detailed medical history data and physical examination, imageological examination is also of great significance in the diagnosis of esophageal foreign body. Simple x-ray plain scan can quickly determine the presence and position of foreign bodies, while CT scan can also determine the size of the foreign body and give an overview of the adjacent structural injuries, which may be critical for establishing a subsequent treatment plan. Low-dose CT scan is considered to be more appropriate for the diagnosis of fishbone incarcerated in the esophagus (3). Currently, endoscopic treatment is commonly preferred in the removal of esophageal foreign bodies with a highly successful rate, but it is still challenging in some cases. We reported a successful case below, in the hope of offering some references for the management of special cases with esophageal foreign bodies. In this case, as the fishbone incarcerated in the esophagus was too large to be removed directly under the gastroscope, we used the holmium laser to break it under the gastroscope before successful removal.

## Case report

**Figure 1:**
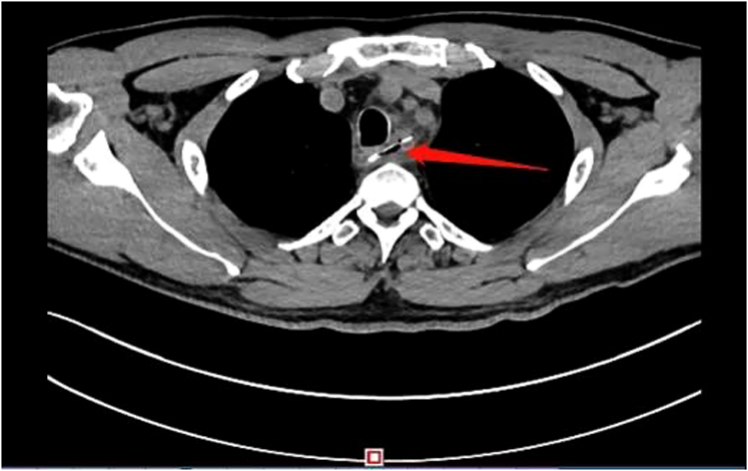
Chest CT showed a hyper-dense shadow of a fish bone shape in the upper Esophagus.

**Figure 2:**
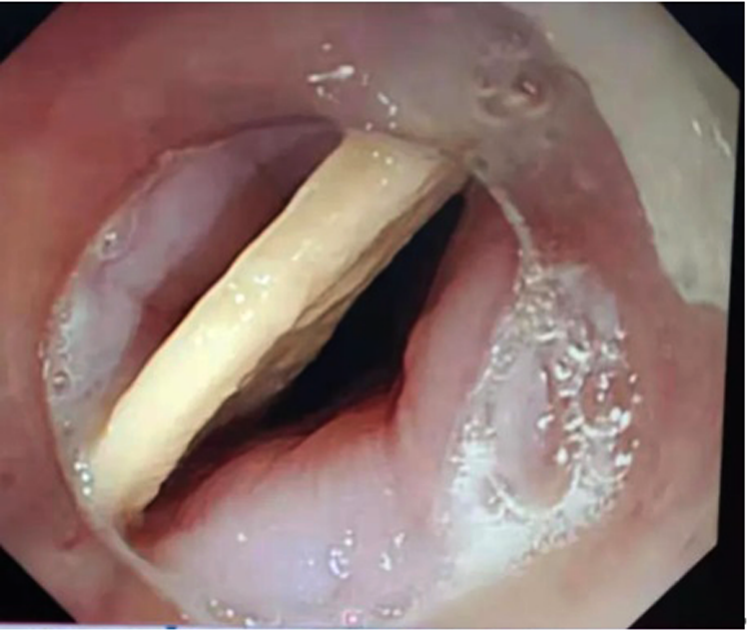
The fishbone was 22 cm away from the incisors with both pointed ends punctured into the esophagus wall.

**Figure 3:**
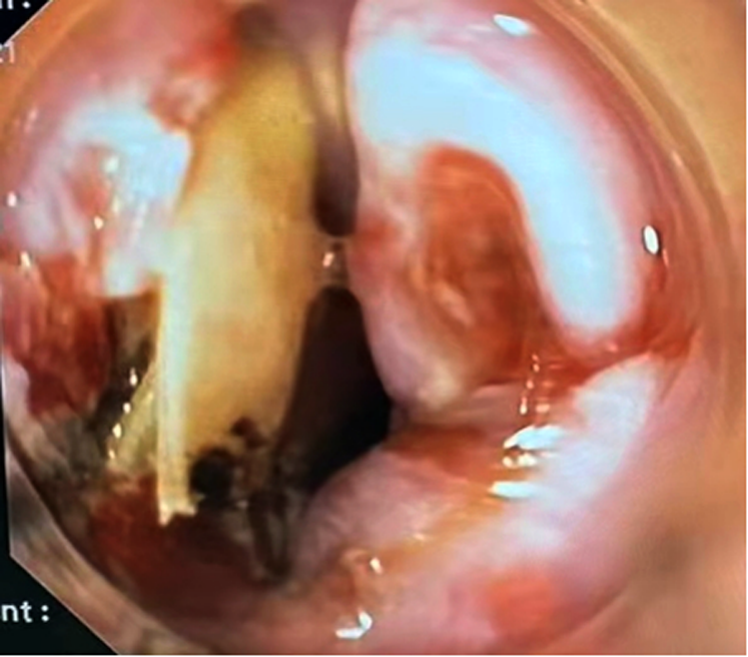
The fishbone was broken by holmium laser endoscopically.

**Figure 4:**
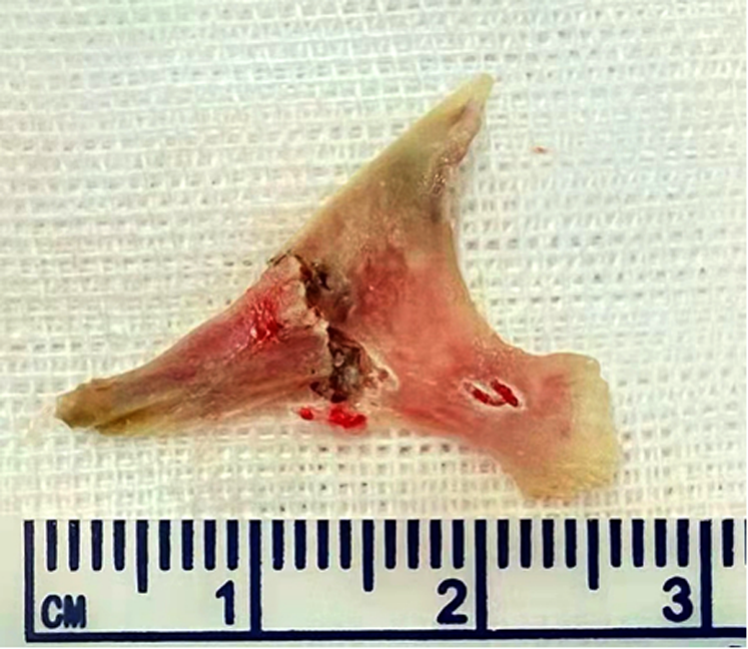
The fishbone was removed after being broken by the holmium laser.

A 51-year-old male patient was admitted for “dysphagia with chest pain for 2 days”. 2 days before admission, the patient presented dysphagia and chest pain without other accompanying symptoms after eating fish. Before being referred to our hospital, the patient accepted a CT examination at a local hospital, which indicated that a foreign body was embedded in the esophagus. The foreign body couldn't be removed under the endoscope because it was large with both pointed ends punctured into the esophagus. He was then referred to our hospital. Previously, the patient underwent coronary stenting due to coronary heart disease and had medical history of hypertension and gout. No obvious abnormalities were observed in his physical examination at admission. Laboratory examination: white blood cell (WBC), 16.34 × 10^9^/L; platelet, 156 × 10^9^/L; neutrophil, 89.5%; hemoglobin, 140 g/L; ultra-sensitive C-reactive protein (UsCRP), 54.1 mg/L; procalcitonin (PCT), 0.116 ng/ml; normal urine routine, stool routine and coagulation function. An irregular hyper-dense focus (transverse diameter: about 2.7 cm; vertical diameter: about 1.7 cm) was observed within the upper thoracic esophagus (T2-T3) on esophageal CT scan, the fat space around which presented patchy and flocculent exudation. In the meantime, the adjacent esophageal wall thickened with rough edges and a small amount of gas accumulated on the left side. The foreign body was only 8 mm away from the aorta, with traces of exudation and gas from the esophagus due to the long retention time of the foreign body (Figure 1). Therefore, it was speculated that the fishbone had punctured the esophagus and caused an infection. Endoscopic removal of the fishbone was attempted at first. However, it was rather difficult and risky, as the fishbone was 22 cm away from the incisors with both pointed ends punctured into the esophagus (Figure 2) and there were manifestations of perforation and infection. After a multidisciplinary discussion, holmium laser was used to break the fishbone into two segments, which were then removed by gastroscopy (Figures 3, 4). A gastrojejunal tube was indwelling during the operation. The patient was fasted postoperatively and then underwent anti-infection (piperacillin-tazobactam + imipenem-cilastatin) and intravenous nutritional therapies. The patient's symptoms of dysphagia and chest pain were relieved after all the efforts. Seven days after the operation, the wound was healed by gastroscopy. Physical reexamination revealed WBC, 4.79 × 10^9^/L; neutrophil, 64.1%; hemoglobin, 128.9 g/L; platelet, 358.1 × 10^9^/L; UsCRP, 0.96 mg/L. The patient was then discharged after resuming the normal diet.

## Discussion

Endoscopic removal of esophageal foreign bodies is of great security and associated with little discomfort with a success rate of over 90%, making it the preferred method for this clinical emergence. Less than 1% of patients will accept surgeries if the esophageal foreign bodies are difficult to remove endoscopically (4,5). Food bolus obstructed in the esophagus can be pushed into the stomach gently, while foreign bodies that are inappropriate or cannot be pushed into the stomach, after the types and positions are identified endoscopically, can be removed by a snare, grasper, stone extraction mesh basket, balloon and other tools. In cases with esophageal foreign bodies, timely management is required, as the incidence of complications will increase notably if the incarceration time is beyond 24 h (6). Otherwise, it will be accompanied by topical mucosal inflammation, ulcer and even perforation, leading to fatal complications such as mediastinum and pleural cavity infections and aortic injury. In addition, the complicating aortoesophageal fistula usually results in massive hemorrhage and secondary infection, making the mortality rate exceed 90% (7).

In this case, the fishbone was long and punctured mediastinum through the esophagus, only 8 mm away from the aorta. In this context, direct removal with forceps might further injure the mediastinum and aorta, triggering severe outcomes. Therefore, we adopted an innovative approach after multidisciplinary emergency consultation. Holmium laser, which is commonly used in minimally-invasive surgeries for urinary disorders and spondylopathy, was utilized in removing the fishbone. With the pulsed emission at a wavelength of 2.1 *μ*m, holmium laser can instantly heat up and shatter objects. Moreover, the holmium laser is still of high security as a great amount of the energy can be absorbed by water and the penetration depth is no more than 0.5 mm, enabling the surrounding tissues to get mild injuries only. In a previous case, a fishbone punctured the bronchus through the esophagus and was removed successfully after being broken by holmium laser bronchoscopically (8). This demonstrates that it is feasible and effective to break the incarcerated foreign body with holmium laser and then remove it endoscopically. In the present case, we removed the fishbone by gastroscopy completely and easily after breaking it with holmium laser, without performing thoracotomy and with few complications. By now, there are various approaches to removing foreign bodies in the gastrointestinal tract, with each one featured by different advantages. In this case, the method of breaking the esophageal foreign body with holmium laser endoscopically is an effective supplement to the commonly used ones and is worthy of further promotion owing to the high security, fewer complications and lower medical expenditures.

## Data Availability

The original contributions presented in the study are included in the article/Supplementary Material, further inquiries can be directed to the corresponding author.
